# A modular strategy for the testing and assessment of non-genotoxic carcinogens

**DOI:** 10.1007/s00204-024-03753-y

**Published:** 2024-05-29

**Authors:** Kimmo Louekari, Miriam N. Jacobs

**Affiliations:** 1 Helsinki, Finland; 2 UK Health Security Agency, Radiation, Chemical and Environmental Hazards, Harwell Science and Innovation Campus, Chilton, Oxfordshire OX11 0RQ UK

**Keywords:** Non-genotoxic carcinogenicity, IATA, Modular strategy, Regulatory framework

## Abstract

A modular strategy is described for the testing and assessment (MoSt) of non-genotoxic carcinogenicity (NGTxC) that is suitable for regulatory applications. It utilizes and builds upon work conducted by the OECD expert group on NGTxC. The approach integrates relevant test methods from the molecular- to cellular- and further to tissue level, many of which have been recently reviewed. Six progressive modules are included in the strategy. Advice is provided for the iterative selection of the next appropriate test method within each step of the strategy. Assessment is completed by a weight of evidence conclusion, which integrates the different streams of modular information. The assessment method gives higher weight to findings that are mechanistically linked with biological relevance to carcinogenesis. With a focus on EU-REACH, and pending upon successful test method validation and acceptance, this will also enable the MoSt for NGTxC to be applied for regulatory purposes across different regulatory jurisdictions.

## Introduction

Cancer is a leading cause of death in rich countries (Dagenais et al. 2020), despite improved therapies and early diagnostics. In a recent global cancer policy review (Madia et al. 2019) observes that:

- The WHO Assembly Resolution, 2017 ‘*urges Member States, […], to implement comprehensive cancer prevention and control programs, including management of disease […] to enhance the coordination of activities related to the assessments of hazards and risks and the communication of those assessments’.* And
- ‘*Cancer is a growing public health concern which requires increased attention, prioritization and funding’*
- United Nations, 2015 ‘*[…] to substantially reduce the number of deaths and illnesses from hazardous chemicals and air, water and soil pollution and contamination’*. (Extracts from Madia et al. 2019)

The current methodology for assessing carcinogenicity hazard mostly focuses on genotoxic carcinogenicity and has not led to a measurable increase in identifying novel carcinogens among industrial chemicals, over the last 15 years (Karamertzanis et al. 2019). This is partly because the number of new in vivo carcinogenicity studies conducted for industrial chemicals is low, and because alternative approaches need to be included.

On the one hand, a portion of unidentified carcinogens is likely to be of low annual production volume, which is subject to reduced information requirements under EU-REACH (EC Regulation No 2006/1907). According to the EUs Chemicals Strategy for Sustainability, ‘*information required for substances in the low and medium tonnages under REACH does not fully allow to identify substances with critical hazard properties. Strengthening information requirements on the carcinogenicity of substances and on other critical hazards at all production levels plays a fundamental role in the successful fight against illnesses such as cancer. … The Commission will amend REACH information requirements to enable identification of all carcinogenic substances manufactured or imported in the EU, irrespective of the volume’.* https://ec.europa.eu/environment/pdf/chemicals/2020/10/Strategy.pdf

On the other hand, for industrial chemicals with high production volumes (> 1000 t/a), a lifetime rodent cancer bioassay (RCB) (OECD Test Guideline (TG) 451) *may* be requested, *if* criteria which demonstrate existing hazard and exposure are met, according to the standard testing strategy. However this is rare for industrial chemicals and environmental toxicants, indeed, very few RCBs have been requested under the EU-REACH Regulation. This is because the legal *information requirement* is conditional and only set at the highest tonnage band (EC Regulation No 2006/1907). The RCB is a standard requirement under many jurisdictions for chemical production sectors, particularly plant protection products, biocides and pharmaceuticals. Whilst there is a concern regarding the adequacy of the RCB [e.g. Rethinking Carcinogenicity Assessment for Agrochemicals Project (ReCAAP) (Hilton et al. 2022)], and alternative approaches are under development for application to many chemical sectors, it is still recognised to have some utility (Suarez-Torres et al. 2021).

Overall, regulatory tools to identify and manage NGTxCs^1^ are poor. This poses a substantial burden to public health. Moreover, there are no internationally agreed specific TGs to address this toxicity endpoint and mode(s) of action of carcinogens. Consequently, there is a need to develop (validated) TGs and testing strategies, on the basis of the current understanding of carcinogenicity and its underlying biology.

In 2016, the OECD established an expert group (EG) to develop an integrated approach to the testing and assessment (IATA) for NGTxC. The vision of the EG is to realistically accommodate different theories and approaches to carcinogenicity hazard assessment and to address all the key hallmarks and key events of the non-genotoxic mechanisms and modes of action that can ultimately lead to cancer outcomes (Jacobs et al. 2016, 2020). The testing and assessment strategy proposed here is based upon the work of the EG. The EG considered that a battery of validated test methods is needed, to address the limitations of the rodent cancer bioassay, together with regulatory strategies to assess NGTxC.

A consensus summary of the key hallmarks of NGTxC (Goodson et al. 2015; Hanahan and Weinberg 2000, 2011), as adapted in Jacobs et al. (2016), and representative tests that address them, was agreed by the OECD EG and published in Jacobs et al (2020). It has been applied as a grouping format to organise assay blocks that address early (molecular) to mid (cellular) to later (tissue) biological key events. These are aligned according to increasing adversity and specificity associated with cancer initiation, promotion/progression and tumour formation. This approach is similar to and consistent with that taken with certain other toxicity endpoint IATAs at the OECD (OECD 2016, 2017a, 2019). Several test methods provide information on these early molecular initiating steps or very early key events (KEs) and later KEs that are available from the scientific literature and assay databases.

Available screening tests for NGTxC are generally not fully validated yet and are not mandatory under any regulatory jurisdiction. For example, ECHA can only request data that is mandatory under the EU-REACH Regulation, and at present that does not include in vitro tests for screening of NGTxCs. Once such screening tests are appropriately validated and internationally accepted by the OECD (see Information box 1 regarding the key concepts in relation to the OECD chemicals regulatory assessment), it will be possible to include them in relevant regulations and different regulatory jurisdictions. This will support the national and regional competent authorities in the identification of likely carcinogens.

Adverse outcome pathway (AOP) thinking has been applied to develop this strategy, by structuring known mechanisms from molecular- to cellular- to tissue level. This approach was used to first build the history of cancer models, which then became the basis of the overarching IATA for NGTxC at the OECD (Jacobs et al. 2020, 2016). In addition, a few endorsed AOPs address Molecular Initiating Events (MIE) such as CYP2E1 Activation leading to liver cancer, AhR and the KE of sustained cell proliferation leading to breast cancer [AOP wiki accessed 07/02/2023 and 15/03/2024, AOPs number 220 AOP-Wiki (www.aopwiki.org), 439 (Benoit et al. 2022)].

Several methods with potential for the identification of NGTxC have been critically evaluated with respect to readiness for TG development (Jacobs et al. 2020). Currently, these focus on pre-screening by utilising gene signalling databases and tools (Oku et al. 2022), combination assay tools that include epigenetic mechanistic assays (Desaulniers et al. 2021), metabolism (Jacobs et al. 2022b) and gap junction mechanistic assays (Sovadinová et al. 2021), followed by the subsequent pivotal module cell transformation (Colacci et al. 2023) and cell proliferation (Strupp et al. 2023). Whilst several assay tools are able to address oxidative stress (a more indirect early mechanism), the mechanisms and AOP pathways are further elucidated by (Veltman et al. 2023), according to the OECD NGTxC IATA (Jacobs et al. 2020). Additional intermediary core key events of inflammation, immune evasion and suppression, plus apoptotic mechanisms, are also proposed (Corsini et al., Vaccari et al., papers in prep).

Although the suitability of non-standard methods for regulatory purposes needs to be judged on a case-by-case basis, and include critically assessed existing information, this evidence should always be considered before designing and conducting in vivo studies. Evidently, guidance in relation to the use and generation of data for NGTxCs will require regular updates as test method development work progresses.

This article outlines an iterative modular approach and integrated assessment of the different elements of existing and newly generated information. This iterative approach may enable decisions on necessary risk management early on or inform on the need for further testing and enable prioritisation.

Information box 1 **International OECD chemicals regulatory hazard assessment**

**Key concepts**

**Mutual Acceptance of Data (MAD) with respect to OECD Test Guidelines **

Reliable and reproducible data is essential to support the international MAD principle for all OECD member country chemical regulatory systems. The data needs to be generated using validated test methods. The MAD framework ensures the generation of high quality and reliable non-clinical test data for regulatory purposes. It was developed in response to fraudulent studies submitted to regulators, by some test laboratories. OECD Principles of Good Laboratory Practices (GLP) provide the quality standards, OECD Test Guidelines (TGs) provide the scientific standards.

Regulatory authorities receiving the data under the MAD know that particular quality and scientific standards were followed, and that they do not have to re-evaluate a test protocol to determine its robustness, as it has consensus by countries via the OECD TG programme, confirmed by scientific experts of the member countries.

OECD TGs and OECD Principles of Good Laboratory Practices are covered by MAD. All other OECD documents are not covered by MAD.

OECD Council Act on MAD (1981) ‘*Data generated in the testing of chemicals in an OECD Member country in accordance with OECD Test Guidelines and OECD Principles of Good Laboratory Practice shall be accepted in other Member countries for purposes of assessment and other uses relating to the protection of man and the environment’.*

Note that data requirements and interpretation of test results are government/regulatory jurisdiction prerogatives. No repeat testing for the same data requirement is needed, however ‘acceptance’ does not automatically mean ‘use’ of data.

**MAD and data requirements**

Although data requirements are countries’ prerogative and MAD is about avoiding repeat testing, if data requirements diverge extensively between countries, MAD will become less useful as data will not be used across countries having different requirements. Therefore, the more compatible/similar data requirements are between countries, the more beneficial MAD will be globally for all stakeholders, from human health and environment protection, to keeping down costs to the chemical industries, and facilitating innovation, to substantially reducing animal testing.

**Data interpretation: what happens in practice?**

Although interpretation of test results is a government prerogative, OECD TGs often integrate transformation of raw experimental data (e.g., through prediction models, data interpretation procedures) to generate a test result that addresses more directly a regulatory need (e.g. identification of a hazard). The data transformation/interpretation procedures implemented in the OECD TG are the outcome of OECD member countries’ agreement to do so, to generate meaningful data, reduce room for (mis)interpretation and maintain a common level playing field. It remains countries’ prerogative to use the stand-alone test result to satisfy a data requirement, or to use the test result with other sources of information, in combination with other level of interpretation or criteria, that meet a country specific regulatory need, to not use that test result if their data requirement cannot be satisfied with that test result (alone or in combination).

Key resource Mutual Acceptance of Data (MAD)—OECD accessed 5/11/2023.

## Structure and main features of the modular strategy (MoSt) beyond the IATA

**Fig. 1:**
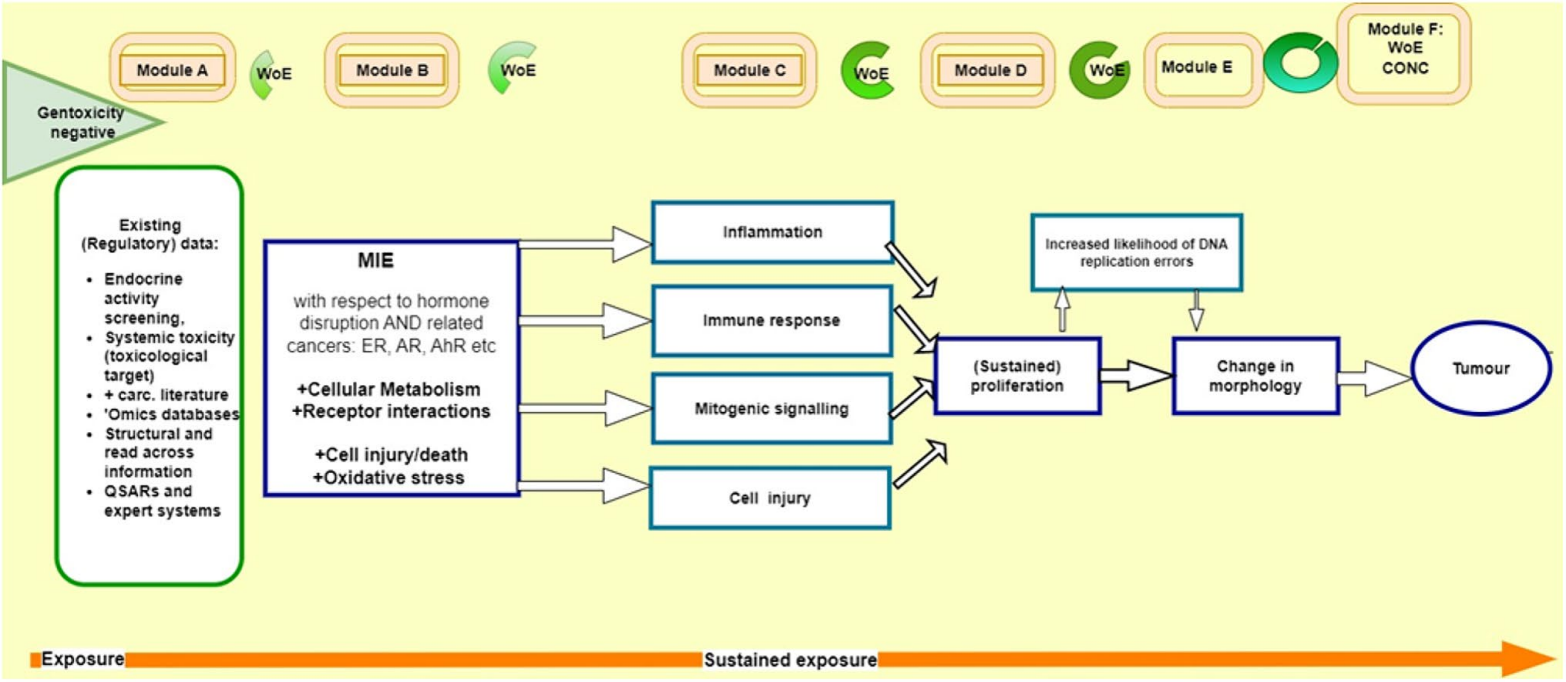
The MoSt: a modular assessment framework for the NGTxC IATA. A brief summary of the MoSt. Each module sits within a box frame populated by relevant assays, including epigenetic and cofactor assay components. Module A consists of an assessment of existing information, utilising also relevant QSAR/in silico tools and ‘omics and other databases. Module B is the MIE module assessing molecular and cellular mechanisms. From module B onwards, many of the modules may be subjected to epigenetic deregulation known to be influential in modulating the specific hallmark module. Module C consists of four pivotal and parallel Key Event (KE) sub-modules that are central to the MoSt. These KEs are not sequential to each other, and can individually, or in combination, lead to (sustained) proliferation. This is further exemplified in Fig. 2. Module D addresses (sustained) proliferation which can increase the likelihood of DNA replication errors also, and Module E addresses a change in morphology. Module F is the Weight of Evidence (WoE) conclusion

For application to the regulatory context, an IATA benefits from being refined into a more defined and stepwise approach, which here is termed a ‘*Modular Strategy of Testing and Assessment*’ (MoSt) for NGTxC. The MoSt proposes how to derive clear step-by-step conclusions for each sequential module, and the generation of relevant new information if necessary. It structures the assessment, explaining how and why conclusions may be drawn for each module. The modular structure of this strategy specifies a decision and assessment steps to support prioritisation and hazard identification (a summary overview is shown in Fig. 1). This testing and assessment strategy is not uniformly applicable in all cases. It is foreseen that application will depend on the strength of the line(s) of evidence that are available for each substance to be assessed. This means that the strong evidence, which supports certain modes of action, should be used to focus and optimise the further testing and the next steps of the MoSt. The strengths and limitations, as well as the potential role of the individual components and tests/assays of this strategy (MoSt) for NGTxCs, are described in detail in references herein.

For expediting the regulatory applicability of the MoSt (and thus the NGTxC IATA), we also integrate the analyses for the critical KEs assessed by the OECD NGTxC IATA expert group, to support the prioritisation of validation studies that need to be conducted. This is particularly needed for the new approach (in vitro and in vivo augmentation) methods (NAMs), aligned with the current EU-REACH information requirements, to technically underpin the regulatory recommendations made.

A further objective of this testing strategy is to determine data gaps in the hazard assessment of NGTxC and how to address these data gaps. This must be achieved whilst making best use of existing in vivo information. The overall aim with respect to chemical hazards is to improve the protection of human health and safety. Minimising the use of animals in the evaluation of carcinogenicity is a desirable concomitant effect, keeping in mind the inherent uncertainties of the RCB (Paparella et al. 2017).

The MoSt is based on mechanisms and modes of action relevant to NGTxC (Fig. 1). It consists of modules, which can be applied sequentially:

- Module A covers assessment of the existing available information and use of existing in silico tools, which can, for example help in formulating a mechanistic hypothesis and therefore guide the selection of the most appropriate tests.
- The next four modules address different NGTxC modes of action and the tests available to investigate these. The modules B, C, D and E follow the order of the corresponding key events in the development of cancer and can detect the relevant modes of action, according to the current understanding of cancer biology.
- In Module F, all the information is evaluated in a Weight of Evidence (WoE) approach.

Weighing the evidence may also be possible in earlier modules, which would shorten the assessment. If such Weight of Evidence (WoE) evaluation is inconclusive, all available information is considered to formulate a hypothesis of the most likely MoA for NGTxC potential of the chemical. This hypothesis will guide the sequence of additional in vitro tests (Modules B, C and D). Thereby, the use of this MoSt can be considered iterative and flexible.

The MoSt is designed to be applied for case-by-case decisions based upon differing degrees of available information:

1. If the WoE is conclusive, a decision on the classification can be made accordingly, depending on the relevant regulatory requirements in the different OECD Member Countries. Most Member Countries (apart from e.g. the USA) adhere to application of the United Nations Global Harmonised System Classification and Labelling of chemicals (UN GHS CL) criteria.
2. When there is strong, almost conclusive information, the selection of further tests can be focused and targeted such that data will be generated only on the crucial mechanistic information that is needed to enable a conclusive decision.
3. When information is scarce, then the screening steps will help in guiding how and why experimental data first needs to be generated.

As soon as new data is available, the results contribute to a new iteration of the WoE analysis and result in a refined outcome of the steps 1–3 above. A methodology to facilitate the WoE evaluation for this endpoint/hazardous property of NGTxC is described in the section on Module F, provided below.

At the next module (B), positive in vitro studies that address the Molecular Initiating Effects (MIE, see Fig. 1) (such as receptor activation, e.g. oestrogen receptor (ER) and androgen receptor (AR) transactivation, OECD TGs 455, 457, 458), and early KE results could contribute to the WoE mechanistic assessment in the context of classification. These studies also give indications of MIE and early KE mechanisms that can guide the conduct of the later and pivotal KEs of cell proliferation and cell transformation in the progression of tumour development. The results of the early KE tests can further guide the selection of the most relevant test method tools and protocols (all of which have different strengths, see for example Strupp et al. 2023; Colacci et al. 2023, for a comparative analysis), to probe the mechanistic basis of the NGTxC action, for these KEs. It is noteworthy to emphasise that while these types of MIE data could support read-across, or also be used within WoE, they are insufficient to trigger the classification on their own. Information gathered from the existing information as generated by the routine 28- and 90-day in vivo studies conducted, and in vivo biomarker data, can be combined to inform upon the selection and design of the subsequent relevant in vitro cell transformation assay (CTA) studies. This is discussed further in the "Possibility to classify non-genotoxic carcinogens" section. The in vitro data can contribute towards the hazard identification when the test results are positive. On the contrary, where first results are negative, further KE screening is necessary.

### Possibility to classify non-genotoxic carcinogens

Classification of a substance as a carcinogen is often based on the balance of positive animal studies and/or epidemiological evidence of the substance causing tumours in the exposed human population. Genotoxicity studies are also considered. Usually, these data are considered in a WoE approach for classification. There are sub-categories of carcinogens which depend upon the strengths and type of the positive evidence that is used for the classification. Under some legislations such as those for pesticides, biocides and food additives, cancer bioassays are normally required and can be used for the decision on the classification. Whilst in the USA, under the National Toxicology Program (NTP) for example, additional selected substances have also undergone animal cancer studies (e.g. Lunn et al. 2022).

It is pertinent to include approaches for triggering and waiving off in vivo studies under specific strong evidential circumstances, as adopted recently by the ICH S1B Addendum and Waivers for Testing Pharmaceuticals for Carcinogenicity https://database.ich.org/sites/default/files/S1B-R1_FinalGuideline_2022_0719.pdf, as well as that described in the retrospective US ‘ReCAAP’ (Hilton et al. 2022).

#### European regulatory requirements

For industrial chemicals (EU-REACH), certain data is generated that can be used in WoE context, when carcinogenicity of the substances is evaluated. These are:

- Existing carcinogenicity studies (the registrants may submit carcinogenicity studies, which have not been requested under EU-REACH, but have been made available)
- Existing epidemiological studies (not available for all substances);
- Genotoxicity studies and
- Sub-chronic toxicity tests, especially the histopathological findings.

Evidence from the repeated dose toxicity studies could influence classification in cases where the data are borderline. As stand-alone tests, these studies cannot at present lead to classification for carcinogenicity under GHS.

Under EU-REACH, the cancer bioassay may conditionally be required. However, this applies only to substances at the highest tonnage level, i.e. manufactured over 1000 tonnes per year and registrant (a single manufacturer or importer) and by demonstrating risk based on available data. More specifically, according to Annex X of the EU-REACH Regulation (Regulation-1907/2006-EN-REACH-EUR-Lex (www.europa.eu)):

‘a carcinogenicity study may be proposed by the registrant or may be required by the Agency …if the substance has a widespread dispersive use or there is evidence of frequent or long-term human exposure, and

the substance is classified as germ cell mutagen category 2 or there is evidence from the repeated dose study(ies) that the substance is able to induce hyperplasia and/or pre-neoplastic lesions’.^2^

For the chemicals registered for EU-REACH, the sub-chronic study data will be available for the chemicals exceeding the limit of 100 tonnes per year per registrant. The observation of hyperplasia and/or pre-neoplastic lesions might be an indication of a non-genotoxic mode of action and are a possible trigger of the cancer bioassay. However, this criterion to request a cancer bioassay has not been applied in a legal decision under EU-REACH to date.

To improve the scope of the data generated, molecular biomarkers of hyperplasia and/or preneoplastic lesions, and for other known later KEs for NGTxC outcomes, such as sustained cell proliferation, can be additional parameters/endpoints that could be implemented by enhancing sub-acute and sub-chronic studies. Adding these biomarkers would not compromise the technicalities and prime goals of these repeated dose toxicity studies. Examples of this are shown in studies by Oku et al. (2022), Strupp et al. (2023), and Colacci et al. (2023).

In the CLP Regulation (Regulation-1272/2008-EN-CLP regulation-EUR-Lex) there are ‘*Some important factors which may be taken into consideration, when assessing the overall level of concern*’ under section 3.6.2.2.6., one of which is:

‘(k) mode of action and its relevance for humans, such as cytotoxicity with growth stimulation, mitogenesis, immunosuppression, mutagenicity’.

This provision can in principle be referred to, in case there is evidence of an NGTxC mechanism. However, most likely this evidence alone would not be sufficient for classification.

For example, a set of 16 chemicals and drugs have been determined to be positive in the CTA SHE assays, and their IARC classification, CLP classification and NTP cancer bioassays have been compared (Colacci et al. 2023). These substances were initially considered non-genotoxic, but later evidence of genotoxicity has been seen for some of them. With respect to this analysis, the CLP classification might appear to be at a lower level, for example some substances have a 2B classification from IARC, but no CMR classification under CLP (although one of these substances has a CLP notification for Carc-2), and a direct comparison between IARC 2B and CLP Carcinogenicity classification is not possible, because the classification criteria differ. Furthermore, about half of the substances in this list are pharmaceuticals, a chemical sector that is not a priority under CLP nor EU-REACH. Annex VI of CLP details what is assessed in the CLH process. For most of these 16 substances, which were initially considered non-genotoxic, a correlation exists between cell transforming properties and in vivo carcinogenesis mechanisms observed in BALB/c 3T3 cells (Colacci et al. 2023). This indicates the utility for the CTA for identification of NGTxCs, within the NGTxC IATA and MoSt. If brought in earlier into the MoSt, essentially run in parallel to the earlier module tests, the CTA may well prove to be a useful confirmatory tool within the overall WoE.

The difference between the classification by IARC and by the CLP committees that apply GHS in Europe is probably mostly due to the different remits of the organisations, the assessment criteria and different information sources. IARC reports are prepared solely on the basis of published data, whilst CLP committees (and the WHO Joint Meeting on Pesticide Residues, JMPR) also receive substantial (unpublished) industry dossier data. Furthermore, IARC tends to classify on the basis of one positive study, whilst CLP and JMPR conduct a WoE analysis of the positive, borderline and negative data. Under CLP, any relevant data, independent of whether it informs upon genotoxic or non-genotoxic mechanisms, can be considered for classification. In terms of classification for carcinogenicity, in vivo data (RCB) or robust human (i.e. epidemiological) evidence is normally necessary. Availability of the latter information is rare. In vitro data on genotoxic or on non-genotoxic modes of action alone would not be sufficient for carcinogenicity classification.^3^ Currently, due to the lack of approved alternative test methods, data generated for non-genotoxic modes of action is lacking. It is noteworthy that harmonised classification under CLP is a resource intensive process, and not all substances of concern have yet been assessed. Without a CLP classification, the risks that these substances pose to public health will not be managed.

### Structure and main features of the strategy: modules A, B, C, D and E

In the first module, Module A, existing information is utilised. In Module B, the molecular initiating events are considered. Module C considers inflammation, immune response, mitotic signalling and cell injury. Module D addresses (sustained) proliferation, and here the essential assay hallmark to be addressed is cell proliferation, triggering investigations on gene and cell signalling and resistance to apoptotic cell death. In Module E, a change in morphology (dysplastic change) represents the point at which adaptive proliferation, hyperplasia, becomes maladaptive. The *change in morphology* module also includes key events of cell trans-differentiation (at the cellular level that is the conversion of one differentiated cell type into another cell type). These include changes in the organisation of the cytoskeleton, acquisition of different morphology and progression to mal-adaptive/irreversible modifications, specifically pathogenic angiogenesis (in contrast to neo-angiogenesis which could be adaptive modifications), genetic instability and then senescence and telomerase activation.

**Fig. 2:**
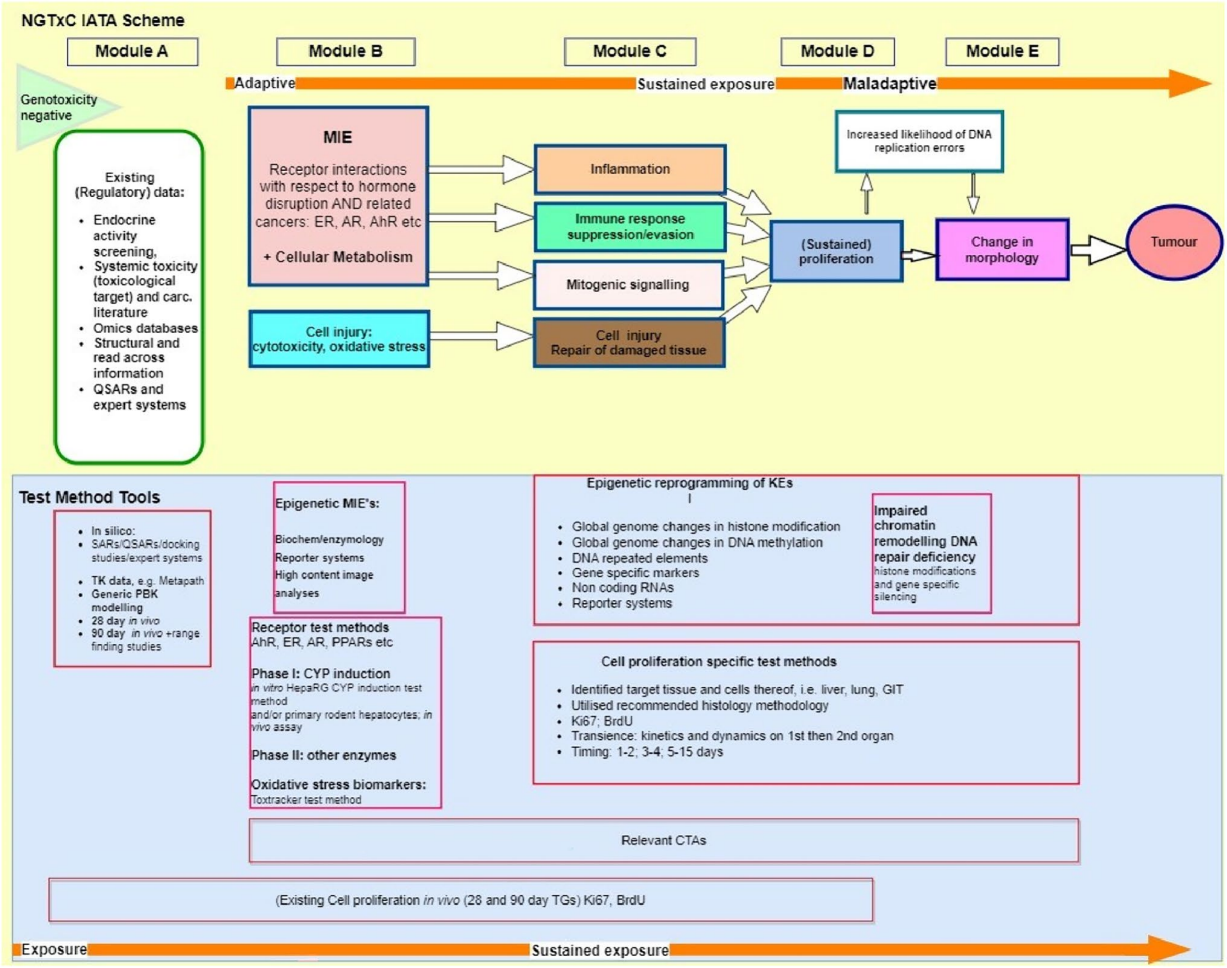
Overview of the MoSt NGTxC (IATA) scheme. The upper part (yellow background) contains the assessment steps and modules corresponding to the different mechanisms involved. The lower part (blue background) summarises the assays and test methods for addressing each specific mechanism and model. The cell transformation assays are considered to be especially useful in the strategy (Colacci et al. 2023), particularly with the use of transcriptomics. This is because they are able to address multiple carcinogenicity mechanisms and biomarkers over different periods of time. They can be used in several relevant mechanistic modules, depending on the preliminary markers identified from earlier key events, MIEs and existing information

Figure 1 above depicts the overarching process identifying the KE stage contribution to carcinogenesis in a sequential approach, whilst Fig. 2 provides further details. The stack of second key events in Module C may require a nonlinear assessment methodology. In the WoE context, the assessment of the evidence may also follow parallel lines of evidence for the Module C modes of action, where one or more of the KEs may be the predominant adverse pathway that triggers Module D, proliferation, and sustains that proliferation.

At this stage, an analysis will need to be conducted with regard to which Module C KE’s contribute to the next modules, or where cell transformation assay tools can be used to confirm the MoA. The CTA can identify the cellular and molecular processes involved in in vitro cell transformation that appear to be the same as those that sustain in vivo carcinogenesis. These processes occur as a result of comprehensive cellular responses to direct and indirect damage to DNA, and thus the CTA is the preferred method to address maladaptive proliferation (Colacci et al. 2023). However, the current, more advanced CTA methods are transcriptomics based and can be used to detect/study the earlier KEs of inflammation, immune disruption, mitotic signalling and cell injury using specific biomarkers, as described by Colacci et al (2023). This pre-screening information could also be obtained earlier in the course of the use of MoSt. Thus, while the CTA is assigned for the late step in the MoSt in Fig. 1, the transcriptomic-based CTA could be applied earlier.

Furthermore, tests can be run in combination, for example the scrape loading test to assess gap junction functionality can be run in conjunction with the CTA (Sovadinová et al. 2021). Gap junction intercellular communication (GJIC) is a fundamental biological cellular process that enables the exchange of various soluble ions and aqueous molecules between adjacent cells, allowing them to integrate multiple signals (e.g. to trigger apoptosis) and coordinate their behaviour. It is a key mechanism for maintaining tissue homeostasis, and its dysregulation has been long recognised as a hallmark of NGTxC. The scrape loading-dye transfer (SL-DT) technique is a simple assay for the functional evaluation of GJIC in various in vitro cultured mammalian cells and can be combined with the CTA. Sovadinová et al. (2021) describe the potential of this assay to be further developed, improved, validated and subsequently utilised as a combination screening/testing tool within the NGTxC IATA and MoSt.

Involvement of the receptor-mediated activation of the metabolic pathways sustaining mechanisms of bioactivation and detoxification of xenobiotics is evident over different time periods, depending upon different test chemical exposure durations. With further characterisation and reproducibility experiments, it will be possible to characterise CTA models for the expression of the receptors that are recognised as the main MIE targets of specific chemicals, and exemplify the specific mechanisms and modes of action (as shown by e.g. Mascolo et al. 2018; Masumoto et al. 2021; Ohmori et al. 2022; Pillo et al. 2022) Masumoto et al. 2021, that play a role in mode and mechanism(s) of action. Whilst the modules are presented as sequential, in practice, early to mid KEs beyond the MIE, before arriving at cell proliferation and cell transformation, especially within Module C, are in parallel.

Nonetheless, careful selection of a CTA based upon the existing information gathered in Module A will support the subsequent selection of the appropriate KE in Module C, such that it will not be necessary to always test in all the modules. The identification of a positive in Module C will be sufficient to move along to Module D. This is already the approach taken for example for genotoxicity in vitro and in vivo testing strategies.

Figure 2 provides a synopsis of the preferred test method and data generation approaches, as explained in greater depth, in the publications referenced in Table 1.

**Table 1:** Correlative mapping of NGTxC mechanisms in relation to the relevant text in EU-REACH guidance (2017) and indication of assay tools and test method readiness status according to the NGTxC IATA (Jacobs et al 2020)

NGTxC mechanisms/(also considered ‘hallmarks’) mentioned in R.7.7.8	Relevant text excerpts from EU-REACH-Chapter R.7a: Endpoint-specific guidance Version 6.0 – July 2017^a^	Key mechanisms and MOA	In vitro mechanistic tools Readiness levels (RL): A = standardised/validated test method/TG, ready for use B = Test method optimised, and validation steps needed C = Test method requires further optimisation and then validation	In vivo mode of action tools	Relevant publications
*(Hormone) Receptor binding (*and transactivation*) modulation of the expression of specific genes*	*… *in vitro* mechanistic studies, …. and (hormone) receptor binding…*	MIE: ER, AR, AhR,	TG455, TG457, TG497, RL: A AhR: ISO guideline in process: RL: B +	Uterotrophic assay (TG 440) and Hershberger (TG441) RL: A	OECD TGs OECD (2018b)
		Steroidogenesis pathway	TG 456: RL: A and augmentation of TG 456 for additional CYP endpoints RL: B		OECD TGs OECD (2018b) EFSA ECHA (2018)
	*…specific receptors (e.g. PPARα, which is associated with liver tumours in rodents; or tumours induced by various hormonal mechanisms)*	PPARα, PPARγ, RAR, PPAR heterodimerisation partner: RXR	(Pre)validation of test methods in Goliath EU H2020 EURION project for PPARα, PPARγ, RL: B Validation underway by the PEPPER platform for PPARα, PPARγ, GR and retinoid receptors RL: B		Legler et al. (2020) OECD (2021) OECD Detailed review paper project no. 4.147 on metabolic disruption in prep
	CYP induction	CYP induction 3A4 (PXR); 1A2 (AhR), 2B6 (CAR)	HepaRG CYP induction test method RL: A-B		Bernasconi et al. (2019), Jacobs et al. (2022b), Pelkonen et al. (2023), Person et al. in prep
*Gap junction*	*As *in vitro* mechanistic studies, ….. gap junction communication…* .. *exchange of growth suppressive or other small regulatory molecules between normal and neoplastic/pre-neoplastic cells through gap junctions is suspected to suppress phenotypic expression of neoplastic potential. Disruption of gap junction function, as assessed by a diverse array of assays for fluorescent dye transfer or the exchange of small molecules between cells, may attenuate the suppression of neoplastic potential by normal cells*	Gap junction cell–cell communication can be combined with CTA	Scrape loading dye gap junction assay: RL: B		Sovadinová et al. (2021)
*Cell proliferation*	‘the animal data… cell proliferation, techniques …*repeated dose toxicity tests: can identify tissues that may be specific targets for toxicity and subsequent carcinogenic effects. Particular significance can be attached to the observation of pre-neoplastic changes (e.g. hyperplasia or metaplasia) suspected to be conducive to tumour development and may assist in the development of dose–effect relationships (Elcombe *et al*. 2002). studies on the induction of sustained cell proliferation: substances can induce sustained cell proliferation *via* compensatory processes that continuously regenerate tissues damaged by toxicity*		BrdU and Ki-67 are the most frequently used methods for the detection and quantification of cell proliferation in vivo and in vitro Serum-free liver mitogen test, CAR assay, DNA synthesis proliferation (BrdU in vitro,14C-thymidine), BRAF inhibitors RL: A	BrdU and Ki-67 are the most frequently used methods for the detection and quantification of cell proliferation in vivo and in vitro RL: A	Strupp et al. (2023)
	*(de)differentiation of the altered or target cells*	Cell proliferation and differentiation have an inverse relationship. Terminal differentiation coincides with proliferation arrest and permanent exit from the division cycle Chromatin-regulating complexes that act in conjunction with transcription factors and determine their activity, i.e. SWI/SNF chromatin remodelers	See entries above for cell proliferation, and also cell transformation transformics assays below		Ruijtenberg and van den Heuvel (2016)
*Oxidative stress*		ROS generation assay	TG 495 (2019) RL: A AOP for NGTxC		Veltman et al. (2023)
*altered growth and death rates*	…*ability to inhibit or induce apoptosis: apoptosis, or programmed cell death, constitutes a sequence of molecular events that results in the death of cells, most often by the release of specific enzymes that result in the degradation of DNA in the cell nucleus. Apoptosis is integral to the control of cell growth and differentiation in many tissues….. inhibition of apoptosis can permit pre-neoplastic/neoplastic cells to escape regulatory controls that might otherwise result in their elimination*…	MIE: Apoptosis, KE: Uncontrolled cell proliferation	TUNEL in vitro method, Widely used. Detection of DNA strand breaks via enzymatic reaction, a late apoptotic event RL: B-C Annexin V in vitro test method for the determination of the apoptotic potential of chemicals, suitable for signs of early apoptosis in a given cell population RL: B-C Caspase 3/7 activation (in vitro). The endpoint is specific to the irreversible final apoptotic execution (activation of ‘executioner’ caspases) RL: B DNA fragmentation assay: calculates the percentage of fragmented nuclei/total nuclei: Late = stage apoptosis RL: B-C	TUNEL in vivo. Widely used. Detection of DNA strand breaks via enzymatic reaction, a late apoptotic event RL: B-C Fluoro Jade in vivo, histopathological detection of apoptosis in repeated dose toxicity studies using H&E staining in vivo RL: B-C	Vaccari et al., in prep
Immunosuppression	….immunosuppression…(p591) …*immunosuppressive activity: neoplastic cells frequently have antigenic properties that permit their detection and elimination by normal immune system function. Suppression of normal immune function can reduce the effectiveness of this immune surveillance…(p590)*	TDAR immunosuppression		OPPTS 870.7800, also reviewed in Lebrec et al. (2014); OECD TG 443 (addressing different life stages) TG 407 and TG 408 RL: A	Corsini et al., in prep
		IL-2Luc	TG 444A (2023) RL: A		
		IL-8Luc	TG442E (2023) RL: A		
		TNFα, JAK-STAT	Omics screening: Interferon and IL-signalling, TNFα, JAK-STAT		Oku et al. (2022)
			CTA’s persistence of interferon and IL-signalling, TNFα, JAK-STAT, (CTA specific), IL-6 can be identified: RL: B +		Colacci et al. (2023), Mascolo et al. (2018), Ohmori et al. (2022), Pillo et al. (2022)
		NK cell/host resistance		ICH (2005), ex vivo TG 407 and TG 408 RL: A	
omics	…*omics techniques*…	Multiple biomarkers for all MIE/KEs in the NGTxC IATA	Omics data bases, CTA	TG 407, 408 443	Desaulniers et al. (2021), Mascolo et al. (2018), Ohmori et al. (2022), Oku et al. (2022), Strupp et al. (2023)
Cell transformation	*…*in vitro* cell transformation assays assess the ability of chemicals to induce changes in the morphological and growth properties of cultured mammalian cells that are presumed to be similar to phenotypic changes that accompany the development of neoplastic or pre-neoplastic lesions *in vivo (OECD, 2006). The altered cells detected by such assays may possess, or can subsequently acquire, the ability to grow as tumours when injected into appropriate host animals…’	MIE’s and KE’s leading to sustained proliferation, cell transformation in relation to neoplastic lesions	SHE: MIE’s of cellular metabolism and receptor mechanisms of AhR signalling via CYP1B1, CYP2E1, epoxide hydrolase 1, GSH transferase, and thioredoxin reductase BALB/c 3T3 has additionally been demonstrated to identify Ugt 1a, and AhR signalling via CYP 1A1, and 1B1 KEs of immune mediated inflammation, mitogenic signalling, cell injury and cell senescence SHE and Bhas 42 CTA KE of mitogenic signalling: identification of many different pathways SHE: MAPK3, MAPK4, MAPK5, Ras oncogene family members and Homeobox 1, as well as NF-kB Bhas 42 shown to identify several (IL-1, IL-2, IL-6, TNFR2) BALB/c 3T3 can identify many (including classical and alternative complement pathway, IFN, IL-1, IL-4, IL-6, IL-9, IL-17, IL-18, TNFR2) KE: sustained proliferation majority of markers identified for the SHE and BALB/c 3T3, all differing between these two models. Bhas 42, integrin signalling and JAK/STAT signalling has been reported to be identified RL: B + Original CTAs validated, but inclusion of transcriptomics needs validation SHE + epigenetics DNA methylation		Mascolo et al. (2018), Ohmori et al. (2022), Guichard et al. (2023), Colacci et al. (2023), OECD (2015, 2017b), Desaulniers et al. (2023), Meier et al. (2023)
Epigenetics….		Multiple biomarkers for all MIE/KEs in the NGTxC IATA	Limited under short-term experiment: Short-term enzymology Commercial enzyme biochemistry assay (cell free = metabolism free) High content image analyses (effect of metabolism) Next-generation sequencing (NGS) based methodologies for DNA or HPTM followed by validation, pathway analyses, and demonstration of affected pathways: Heritable epigenetic memory/reprogramming (Long term > 3 weeks) Sat-α DNAm: a more targeted endpoint with larger dynamic range than L1 and AluYb8 (genomic instability). H3K9me2/3 (heterochromatin to euchromatin) H4K20me3 (dysfunctional DNA repair) RL: B	NGS-based methodologies for DNA or HPTM followed by validation, pathway analyses, and demonstration of affected pathways Heritable epigenetic memory/reprograming (Long term > 3 weeks) RL: B miRNA RL: B	Desaulniers et al. (2023, 2021), Hwang et al. (2020), Marchetti et al. (2023)

^a^Direct quotes from the EU-REACH Guidance. The version dated 2017 includes text published in 2007

### Structure and main features of the strategy: module F––weight of evidence

WoE approaches are widely used in regulatory toxicology, e.g. to characterise the hazardous properties, to meet regulatory information requirements, and in classification under CLP, as discussed in the “Possibility to classify non-genotoxic carcinogens” section. WoE assessment is used when there is no single definitive study that can sufficiently address the toxicity endpoints and meet specific legal information requirements. Instead, there may be several studies or data that address the relevant toxic property. According to Annex XI, Section. 1.2. of the REACH Regulation ‘*There may be sufficient weight of evidence from several independent sources of information leading to the assumption/conclusion that a substance has or has not a particular dangerous property, while the information from each single source alone is regarded insufficient to support this notion’.* The sources of information may be in vivo or in vitro studies, QSARs, epidemiological studies, etc. (However, since reliability and relevance should be addressed under WoE assessment, it may happen that those sources of information that do not meet those criteria will not contribute to the final assessment.) We recommend that the scientific evaluation of a WoE adaptation includes:

- evaluation of each source of information provided for (1) **reliability** and (2) **relevance** to establish whether or not each individual source of information can be used within WoE, and
- evaluation of (3) **consistency** and (4) **completeness** of the studies/data can be done in integrative phase, after each piece of information has been addressed (ECHA 2011) (https://echa.europa.eu/documents/10162/17235/information_requirements_r4_en.pdf/d6395ad2-1596-4708-ba86-0136686d205e).

There are formalised ways to assess and record the **reliability **of toxicological studies, e.g. Klimisch scoring for experimental data and Hills criteria for epidemiological studies. Furthermore, adherence to the OECD TGs and GLP give an indication of adequate quality. Since there are no OECD TGs as yet for NGTxC endpoints, one could consider the documentation and the validation status of the test protocol when assessing the quality of a study/assay. Also, the study report may give indication of well-documented test protocols, repeatability within the laboratory, adequate use of positive and negative reference substances, etc., and this would provide a qualitative assessment of the test results.

**Relevance **of a study means that the study results address an effect that is relevant for humans. With respect to NGTxC, the key events and respective assays, summarised in Fig. 2, are considered to be mechanistically relevant. Obviously, using cell lines that are mechanistically human relevant increases the relevance of the data obtained. After reliability and relevance of each study is considered and recorded, each line of evidence needs to be integrated, together with an uncertainty analysis, to reach a conclusion.

For identification of NGTxCs, in principle, all relevant key events should be covered, to achieve **completeness** of the testing strategy. However, in Module C, *parallel* mechanistic events are addressed. Not all of them need to be covered in case there is sufficient overall evidence that one specific sequence of key events has been observed/confirmed. For example, this means that in case there is positive data on initial key events (Module B), positive results from specific immunotoxicity assays **and **confirmatory data from cell transformation and cell proliferation studies, the overall assessment is likely to consider that the dataset is sufficiently complete. In that case, the rest of the key events in Module C do not necessarily need to be addressed.

**Consistency** means that in different studies/data, similar toxicological effect(s) is observed. Thus, consistency of evidence supports the WoE adaptation. On the contrary, a clear inconsistency, in particular when adverse effects are observed in one study, and not observed in another (that covers the same scope) without a clear explanation, would decrease the confidence on the WoE. For NGTx carcinogenicity, even if there is positive data from Modules B and C, but only negative results from CTA and cell proliferation studies, the WoE assessment would be hampered by inconsistency with respect to the later KE data. Therefore, the test substances would be considered as not NGTxC.

In the testing strategy suggested here, WoE analysis would take place after all Modules in Fig. 1 have been addressed/completed. However, this MoSt is a flexible tool in this regard, and WoE assessment may lead to robust conclusions already earlier, based on findings in Module/s A to E:

(A) Existing information and appropriate computational tools are used. In some cases, the computational tools may reduce uncertainty when combined. In a minority of cases, the existing information could be conclusive for classifying a carcinogen. Examples may include that indications in existing data support a grouping- and read-across approach while not sufficient by themselves for classification. The hazard from a structurally similar, known carcinogen could be read-across.
(B) (A + B) A molecular initiating event is required as the starting point in carcinogenesis. At present, outcomes from test methods associated with molecular initiating events are not sufficient on their own, in addition to information from Module A, to lead to classification. Exceptions are those noted under (A). See Information box 2.
(C) After initiation, carcinogenesis requires that at least one of the four pivotal and parallel key events in Module C is present to progress the disease. Positive results from more than one key event in this module increase the confidence that Module C is positive; it would not add to the weight of the evidence in a quantitative manner, that means, cannot replace, e.g. positive findings in other modules. At the early implementation stages of the MoSt, positive findings in Modules (A,) B and C may serve to trigger further testing. They may also contribute information for the detection of other hazards, such as immunotoxicity and endocrine or metabolic disruption without ultimately leading to carcinogenesis.
(D) The sustained proliferation in Module D is a more specific and biologically more relevant finding for carcinogenicity. That, in addition to positive findings in Modules (A,) B and C, would be a stronger indication that a substance may prove to be carcinogenic, and may serve to trigger further testing including higher-tier (in vivo) (Furihata and Suzuki 2023)^4^ testing in early stages of implementation (see Information box 2 ‘*Role of grouping …’*).
(E) Specificity of the evidence increases further with changes in cell morphology in Module E. In addition to positive findings in Modules (A,) B to D, this would lead to a (strong) conclusion that a substance is likely to be a carcinogen and support subsequent classification.

To confirm applicability of the MoSt, testing of known carcinogens may serve to quantify its sensitivity and identify false-negatives and identify which of the discrete test methods have the highest accuracy (see Information box 2 regarding the utility of the role of grouping and read-across).

Conclusions drawn after Modules A and B will often be useful to decide upon further testing needs. For example, careful selection of a CTA, based upon the existing information gathered in Modules A and B, will support the subsequent selection of the appropriate KE in Module C, such that it will not be necessary to always test via all test methods in all the modules. The identification of a positive in Module C will be sufficient to move along to Module D. This is already the approach taken for example for genotoxicity in in vitro and in vivo testing strategies, e.g. under the REACH Regulation: positive findings in either one of two in vitro gene mutation tests, or chromosomal aberration tests, trigger confirmatory studies in vivo which can be specific to the observed mechanism of genotoxicity. Crucial mechanistic information guides the next steps in the testing strategy for genotoxicity, with testing for gene mutations versus chromosomal aberrations (aneugenicity and clastogenicity).

Whilst the focus of this discussion so far has been on NGTxC mechanisms, also mechanistic information on genotoxicity may and should be included in the WoE assessment, e.g. in Module B.

Conclusions drawn within or after Modules C and D will be useful especially when the (intermediate) aim of testing is priority setting or screening, rather than (ultimate) full characterisation on the hazardous property. When the aim is to reach a plausible conclusion as to whether the test substance(s) is/are positive or negative for NGTxC, the WoE assessment would be done after Module E. At that stage, the full range of tests/assays that address the most relevant modes of action have been performed. It is anticipated that for many chemical/substances, the WoE conducted at these stages will either (i) suggest that the substance is likely to be negative or (ii) that the substance is likely to be a (non-genotoxic) carcinogen, especially in the case where the mechanistic hypothesis has been confirmed.

In summary, results from Modules B to E:

- progressively inform upon the need for further testing, with information needs decreasing as one progresses from B to E
- will allow prioritisation of substances, or ultimately their classification/risk management, increasing as one progresses from B to E. As discussed below, regulatory information requirements and classification criteria need to be amended, to allow the adequate use of (validated) in vitro methods and testing strategies.
- support grouping and read-across cases. In time, the database with information from new methods will grow. The experience that assessors gain will build greater confidence in these methods, such that regulatory decisions will also be able to be increasingly based upon the results generated by grouping-approaches (Information box 2) and (validated) new methods within the MoSt (see Information box 2, * Role of grouping...*).

Further advice on how new methods in addition to conventional (short-term) hazard information supports read-across and grouping approaches is available, e.g. in ECHAs read-across assessment framework RAAF and ECHA guidance R.6.

To assist in the evaluation of the weight of evidence, a radar graph approach may be utilised (vom Brocke paper in preparation). Although different, the radar graph tool has visual similarities to pie graph approaches, e.g. *ToxPi* as used by NIHS and described in (Marvel et al. 2018). A decision tree approach can also be utilised.

Furthermore, the integration of results that visualise a biological continuum can reduce uncertainty regarding the Point of Departure’ (PoD) as one moves from MIE to subsequent KE testing. It is also recognised that not all the in vitro test methods within the IATA are currently optimised such that they can provide concentration responses that can be utilised as potency scores (Jacobs et al. 2022a, b). Instead, they are optimised for conventional assessments, which require binary test outcomes that have been obtained from outcomes of the biological continuum (similar to, for instance, the number of revertants in the Ames assay, or stimulation indices in skin sensitisation assays).

In the final weighing of the evidence, a substance that has a high ratio of positive results, with coverage of all the KEs, is likely to be a (strong) carcinogen. To include all stages of carcinogenesis initiation, promotion and tumour progression, information from Modules A and B can and must be included as well. Of course, a substance that is covered by all tests and exceeds the respective pre-defined thresholds has a higher priority for regulatory action, than one that does not.

The MoSt needs to be very clear and transparent with clear demarcation between, e.g. weak positive and positives as well as borderline and equivocal. A simple and more obvious analogy for the development of such a WoE decision approach can be drawn upon current practice in genotoxicity and its quasi-linear decision approaches.

Information box 2 **Role of grouping and read-across in increasing the acceptance of new methods**

During a transition phase, where confidence in new methods is reduced due to limited experience in application, additional (mechanistic) information from tests in Modules B-E may be valuable for supporting cases for grouping and read-across, leading to increased regulatory impact, than if the new methods were considered in isolation.

The quantity and quality of additional information needed depends on the robustness of the grouping and read-across approach: for cases with higher confidence from existing supporting data, fewer additional mechanistic information will be necessary, and vice versa.

With time, assessing the performance of new methods in addition to conventional in vivo data will identify those new methods that allow classification also in the absence of any RCB data. This can support the identification of methods that will contribute to future regulatory landscapes (e.g. Landsiedel et al. 2022).

### Outcome of module F, the weight of evidence assessment

IATAs that are accepted by the OECD (such as those for the skin and eye irritation/corrosion and skin sensitisation) typically have one or more WoE steps included. The WoE assessment that takes place after most KE-related tests have been performed, which aims to answer for example the following questions:

- Is the evidence sufficient for classification?
- Is the evidence sufficient for priority setting?
- Which further tests would be the most pertinent to further characterise the substance?
- Does the evidence strongly suggest that the substance does not have the hazard property in question (non-genotoxic carcinogenicity in this case)?

In summary, with both a decision tree and radar graph approach, biologically related results can be mapped to consolidate both direct and indirect mechanistically related findings. These approaches can portray the weight of their contributions and the added weight from biologically linked results that can be used to examine the test results in a WoE assessment. For example, whenever there is a MIE/KE that is confirmed by one of the mechanistic assays (e.g. receptor activation/immune/gap junction) and further confirmed by a specific CTA, or cell proliferation findings, we will have data that are mechanistically interlinked and indicate a stronger WoE. This will assist the communication and facilitate discussion on the overall findings, to compare different substances and to confirm or refute mechanistic hypotheses on the non-carcinogenic path or sequence of key events.

### Selection of further tests within the strategy

It is foreseen that when this modular strategy is used, at the outset, there will be data suggesting that the substance may have NGTxC properties and/or there is a regulatory interest to further characterise the hazards that the substance may pose to the public health. Looking at the schematic presentation of the MoSt (Fig. 1), there may be several decision-making steps involved when the MoSt is applied. Although these steps may be individual and specific to the case at hand, there are the common elements involved at these steps. As shown in Fig. 2, mechanistic hypothesis and screening data should be taken into account when further tests are considered. Where there is already some specific test data available, these will guide the selection of the next test. Where the positive results/evidence are already considered sufficient––for a given regulatory purpose––no further testing is necessary.

**Fig. 3:**
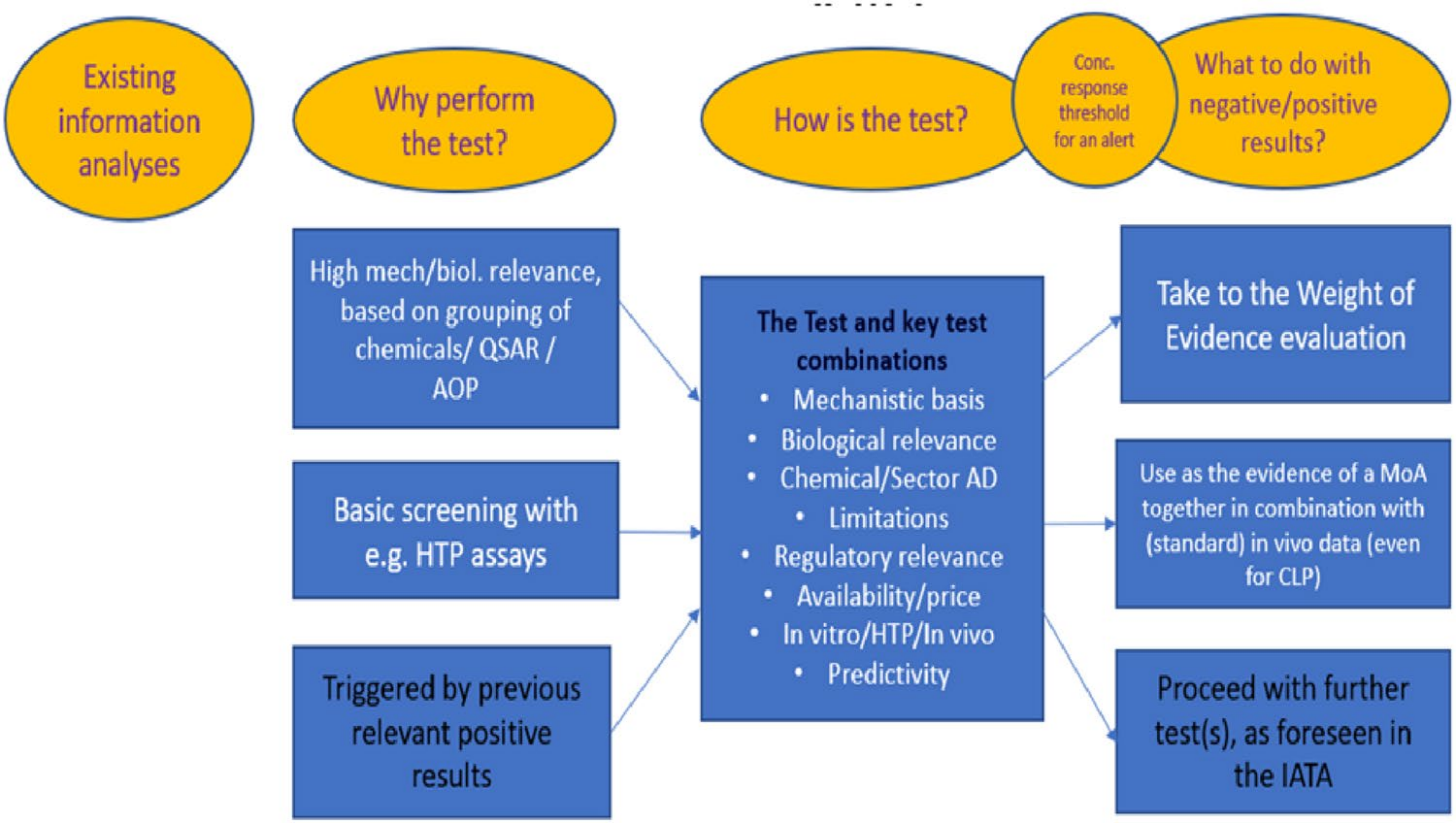
Making a decision on further testing needs and proceeding to the consequent next step(s)

When there are several pieces of evidence and the data is contradictory, a WoE analysis should be performed. While one example of a formal WoE approach using the radar graph approach is given in the previous section, there may be other leaner approaches that can be taken on the basis of the strength of the data available and the complexity of the hazard endpoint. In any case, it is suggested that the WoE analysis is well documented, especially at the final step of the MoSt. As pointed out in Fig. 3, analysis of existing data and test results may also lead to further tests, especially if we are in Module C and pivotal mechanistic information is still missing.

It is foreseen that the MoSt will require the identification of what is adequate and sufficient information for categorising chemicals as carcinogens. This may require weighing the various assay blocks in a different manner. For example, it is probably not necessary to ‘prove’ metastasis steps but stop at the point where adequate prediction of adversity can be achieved to the satisfaction of stakeholders, including industry, regulatory bodies and non-governmental organisations. With respect to chemicals identified as immunosuppressive agents, it may not be necessary to run the chemical also in a CTA, as such substances will not directly transform cells but will decrease immunosurveillance of carcinogenic infectious agents. Whilst with respect to immune-mediated inflammatory responses, the CTA will be highly relevant. We will then apply the uncertainty analysis approach specifically developed for the assay evaluation and the development of the MoSt, having assessed and ranked the assays together with WoE assessment. The next steps will be to conduct further validation work where needed, to generate the data, including point of departure data, to be able to refine the MoSt and develop decision trees.

### Structure and main features of the strategy: further optimisation

Selected assays will be subject to the development, optimisation and (pre)validation within the Test Guideline Programme (TGP) of the OECD. The MoSt as a whole needs to be human relevant and clear about which tests are appropriate for specific KEs. Funding initiatives will then be able to target selected assay validation for TG use within the MoSt. Furthermore, concentration response information is essential for establishing points of departure, although this information from validation studies is not always sufficiently available (see e.g. Jacobs et al. 2022a).

## Forward look

It is foreseen that member countries of the Test Guideline Programme of OECD, and relevant EU projects for example PARC, will pursue the further optimisation and validation needs of the methods that address the most relevant endpoints/hallmarks of cancer. The priorities of the NGTxC test method needs and guideline development have been addressed in Jacobs et al. (2020) and the articles included in Table 1. Furthermore, the test guidelines will be accompanied by an OECD Guidance Document, to give a flexible, modular testing strategy, adaptable for different international regulatory needs. In the OECD NGTxC IATA EG, work is in progress for such a Guidance Document.

After the applicability and regulatory relevance of new test methods have been analysed according to accepted test method readiness criteria, the studies could be considered for inclusion in the lower tonnage information requirements of EU-REACH.

In the TG projects selected and agreed by the OECD Working Group of National Coordinators to the Test Guideline Programme, the conduct of proper validation studies is a crucial element. Data and the resources for validation studies are not easily obtained, and require dedicated funding, time and experienced management.

Industries do have a lot of relevant data that could be used, e.g. to include positive and negative controls and to list adequate reference substances for the validation studies. One mechanism to facilitate the data acquisition for the retrospective validation studies is the so-called honest broker, or neutral broker approach. Here, a neutral (independent, with no vested interests) expert or party receives the anonymised data from industry, collates and communicates it to the validation management team participants of the study and ensures the confidential handling of such data. This mechanism has been previously applied to both in vivo and in vitro studies, as seen for example with respect TG 433 (Acute Inhalation Toxicity: Fixed Concentration Procedure) and revisions to the TG 456 (the steroidogenesis assay). Whilst we are keen to encourage industries and CROs to contribute the confidential information that they have generated as relevant evidence for such validation purposes, it is important to also protect their commercial sensitivity and interests.

Optimally, the validation studies would demonstrate acceptable levels of sensitivity and specificity of a method. From the public health point of view, sensitivity of methods is crucial because positive results would normally lead to enhanced regulation and risk management. Therefore, identifying substances that are positive for (non-genotoxic) carcinogenicity is important, whilst ascertaining that a substance is not an NGTxC does not require regulatory action. On the contrary, it is in the interest of society and the chemical industry that also the number of false positive results is minimised, to facilitate green chemistry. Assessment outcomes that are ‘positive for toxicity to humans’, are legally easier to turn into regulatory actions. Indeed, relevant existing regulations are based on this principle (e.g. CLP).

Chemicals of particular interest in the design of suitable validation studies for the NGTxC IATA KE’s can be derived from many of the preliminary chemical lists in the relevant tables in Colacci et al. (2023), for the CTA, and Strupp et al. (2023) for cell proliferation. Furthermore, representative coverage of appropriate chemical applicability domains for which the regulatory test method is intended is needed, and ideally the chemical selection will be developed in an integrated manner, keeping in mind the marker mechanisms identified thus far for both the late and early KEs in the IATA. For instance, capturing the early apoptosis and late apoptosis stages, the early cell proliferation and late-stage proliferation, and both the initiation and promotion in the different CTAs, needs to be a critical part of this discussion.

Supporting information with respect to suitable reference and proficiency chemicals can also be obtained from animal bioassays, and from an adequate epidemiological setting. OECD Guidance Documents on good in vitro practice guidance (OECD 2018a, b) and validation (OECD 2005) and several literature publications in addition to the papers in Table 1 describe in more detail the process by which well-evidenced negative and positive chemicals for validation studies should be derived.

Parallel to these OECD TGP projects, application to regulatory guidance and potential revisions can be considered. Under different sets of legislations, guidance has been provided on how the hazard information should be assessed. One example of these is EU-REACH ‘Guidance on Information Requirements and Chemical Safety Assessment’ The chapter on Carcinogenicity (R.7.7.8) Guidance on IR&CSA––Chapter R.7a (europa.eu): this EU-REACH Guidance is intended to help the registrants generate and obtain relevant information on the substances to assess the hazards that they may pose to human health. The description is as follows: ‘*The process of carcinogenesis involves the transition of normal cells into cancer cells *via* a sequence of stages that entail both genetic alterations (i.e. mutations) and non-genetic events. Non-genetic events are defined as those alterations/processes that are mediated by mechanisms that do not affect the primary sequence of DNA and yet increase the incidence of tumours or decrease the latency time for the appearance of tumours. For example; altered growth and death rates, (de)differentiation of the altered or target cells and modulation of the expression of specific genes associated with the expression of neoplastic potential (e.g. tumour suppressor genes or angiogenesis factors) are recognised to play an important role in the process of carcinogenesis and can be modulated by a chemical agent in the absence of genetic change to increase the incidence of cancer.*

Table 1 identifies the relevant mechanistic text from the current EU-REACH guidance and correlates it with the test method review work conducted within the programme of work in the OECD NGTxC IATA EG and the endpoint/assay reviews that the EG are conducting. Among the relevant in vitro studies, the cell transformation assay and studies on altered intercellular gap junction communication are included, as well as studies on hormone receptor or other receptor binding and transactivation, also inhibition of apoptosis, from early to late stages, and immunosuppressive and evasive activity. All the CTA models can identify various different cell injury mechanisms, but only the SHE CTA and the Bhas-42 CTA have been shown to pinpoint senescence bypass and telomerase signalling. The CTAs are able to identify the markers indicated in Table 1, for the sustained proliferation that can alter the tumour microenvironment, and then also the cell adhesion and cytoskeleton, which together can lead to oncotransformation. Epigenetic markers can be identified from early to late stages of the IATA, with a variety of suitable tools, including next-generation sequencing (NGS), but these require further optimisation with a larger set of potential reference chemicals for NGTxC and subsequent validation to be suitably standardised.

As shown in Table 1, the correspondence between EU-REACH and the MoSt presented here is good, which can be regarded as a positive signal for the test developers, the OECD community and the legislators.

Thus, it is already recognised in the ECHA guidance that these types of data could be used in the assessment, for the hazard identification. However, where the Guidance addresses the data requirement for hazard identification and classification, only cell transformation is mentioned. This is due to the recent lack of validated test methods and lack of specific legal information requirements. Now the priorities for taking forward the validation needs are identified for cell transformation (and consolidated in Table 1, where readiness criteria indicate level B: optimised, but interlaboratory reproducibility validation now needs to be demonstrated). The work of the OECD EG will be a useful contribution to any future plans for an update within the ECHA guidance. Whilst the basis of addressing non-genotoxic carcinogenicity has been laid out and clearly indicated in the EU-REACH Guidance, we can now address and remediate the identified ‘*shortage of sensitive and selective test systems to identify non-genotoxic carcinogens, apart from the carcinogenicity bioassay’.*

Here, we have taken a pragmatic approach for utilising the critical review work conducted by the OECD EG developing an IATA for NGTxC. We utilise existing information extensively, looking at how to apply tests that are already being run and how to improve the quality of the data being generated from them. Existing test methods (e.g. OECD TG407 and 408) can be enhanced, modified or combined to be useful for obtaining critical information on NGTxC, in particular for the KE of cell proliferation. Some of the test methods may have (un)expected cost implications. It is important to maximise and optimise the use of existing and regularly applied test methods.

Classification of substances for non-genotoxic carcinogenicity has been addressed above. After the relevant assays are formally considered for approval by the OECD TGP, the regulatory agencies and industry––in the case of self-classification––might appreciate more detailed and more comprehensive advice on the use of the test data. It would be unfortunate if the regulators would find that legal provisions and guidance do not need to be revised because there are no relevant approved TGs yet, whilst the TG community might think that developing the guidance is of low priority because there is no regulatory use of those test data. The NGTxC IATA test method validation priorities are itemised under Readiness Level (RL) B in Table 1, and in line with the OECD call for the validation of regulatory applicable test methods (https://web-archive.oecd.org/2023-01-23/650072-urgent-mobilisation-national-regional-resources-to-support-the-validation-of-new-methods-safety-testing-of-chemicals.pdf), funding bodies are encouraged to address funding these needs in their funding calls, going forward.

Parallel activities and dynamic exchanges are needed, and this is being considered in for instance EU-funded projects such as PARC (Audebert et al. 2023; Marx-Stoelting et al. 2023), where, going forward, test method developers and regulators have the opportunity to work closely together. Robust comparative and combinatorial analyses of carcinogenicity computational tools are not new, they have been conducted for over 20 years, e.g. Lewis et al. (2002) and many are publicly and commercially available (e.g. OECD QSAR toolbox https://www.oecd.org/chemicalsafety/risk-assessment/oecd-qsar-toolbox.htm accessed 15/03/2024) and (Cayley et al. 2023), respectively). Similarly, comparative analyses of the more recent biomarker databases can support the development of additional regulatory tools and their application for carcinogenicity in Module A, existing information, as shown for example by (Oku et al. 2022). Applications for error-corrected next-generation sequencing (ecNGS) currently being developed for genotoxicity are also promising for NGTxC (Marchetti et al. 2023).

An immediate need will be to achieve consensus on chemicals selected for the further validation of the priority test methods for the different KEs and recommendations as to how to practically design the validation studies to optimise the IATA and the regulatory modular approach. The methodology presented here is flexible and includes the mechanistic links of biological relevance between KEs to be integrated into the assessment outcome. It enables the assessment of complex hazard endpoints where KEs are likely to occur in parallel, or in a network.

## Data Availability

Not applicable.

## Footnotes

1 The previously agreed definitions of genotoxic and non-genotoxic carcinogenicity are: ‘*The induction of cancer involves the accumulation of genomic alterations, which can be induced directly or indirectly. Carcinogens have conventionally been divided into two categories according to their presumed mode of action: genotoxic carcinogens and non-genotoxic carcinogens. A genotoxic carcinogen has the potential to induce cancer by interacting directly with DNA and/or the cellular apparatus involved in the preservation of the integrity of the genome. A non-genotoxic carcinogen has the potential to induce cancer without interacting directly with either DNA or the cellular apparatus involved in the preservation of the integrity of the genome Jacobs MN, Colacci A, Corvi R, *et al*. (2020)*’.

2 “*If the substance is classified as germ cell mutagen category 1A or 1B, the default presumption would be that a genotoxic mechanism for carcinogenicity is likely. In these cases, a carcinogenicity test will normally not be required.”.*

3 In the future, GHS may be revised, in order to better enable the use of in vitro data on non-genotoxic MoAs for classification. However, before that potential revision, there needs to be a good data evidence base showing that the in vitro methods for non-genotoxic carcinogenicity give a reliable prediction. It is also recognized that revision of the GHS criteria can be a protracted consensus process, and is unlikely to occur in the short term.

4 Short-term in vivo methods that could contribute to definitive information at this phase are proposed and used in the pharmaceutical sector (see for e.g. Furihata C, Suzuki T (2023), and reviewed in Committee on Carcinogenicity of Chemicals in Food Consumer Products and the Environment (COC) (2019) Statement COC/G07—Version 1.1 Alternatives to the 2-year Bioassay.).

## Declarations

### Disclaimers

Kimmo Louekari (retired) was a staff member of the European Chemicals Agency until September 2021. Both the authors are members of the OECD NGTxC IATA expert group. The views and opinions expressed in this article represent exclusively the personal ideas of the authors and do not represent the official position of the  European Chemicals Agency or the UK Health Security Agency.
